# SMA-Causing Missense Mutations in *Survival motor neuron (Smn)* Display a Wide Range of Phenotypes When Modeled in *Drosophila*

**DOI:** 10.1371/journal.pgen.1004489

**Published:** 2014-08-21

**Authors:** Kavita Praveen, Ying Wen, Kelsey M. Gray, John J. Noto, Akash R. Patlolla, Gregory D. Van Duyne, A. Gregory Matera

**Affiliations:** 1 Curriculum in Genetics and Molecular Biology, University of North Carolina, Chapel Hill, Chapel Hill, North Carolina, United States of America; 2 Integrative Program for Biological and Genome Sciences, University of North Carolina, Chapel Hill, Chapel Hill, North Carolina, United States of America; 3 Department of Biology, University of North Carolina, Chapel Hill, Chapel Hill, North Carolina, United States of America; 4 Department of Biochemistry & Biophysics, University of Pennsylvania, Perelman School of Medicine, Philadelphia, Pennsylvania, United States of America; 5 Lineberger Comprehensive Cancer Center, University of North Carolina, Chapel Hill, Chapel Hill, North Carolina, United States of America; The Jackson Laboratory, United States of America

## Abstract

Mutations in the human *survival motor neuron 1* (*SMN*) gene are the primary cause of spinal muscular atrophy (SMA), a devastating neuromuscular disorder. SMN protein has a well-characterized role in the biogenesis of small nuclear ribonucleoproteins (snRNPs), core components of the spliceosome. Additional tissue-specific and global functions have been ascribed to SMN; however, their relevance to SMA pathology is poorly understood and controversial. Using *Drosophila* as a model system, we created an allelic series of twelve *Smn* missense mutations, originally identified in human SMA patients. We show that animals expressing these SMA-causing mutations display a broad range of phenotypic severities, similar to the human disease. Furthermore, specific interactions with other proteins known to be important for SMN's role in RNP assembly are conserved. Intragenic complementation analyses revealed that the three most severe mutations, all of which map to the YG box self-oligomerization domain of SMN, display a stronger phenotype than the null allele and behave in a dominant fashion. In support of this finding, the severe YG box mutants are defective in self-interaction assays, yet maintain their ability to heterodimerize with wild-type SMN. When expressed at high levels, wild-type SMN is able to suppress the activity of the mutant protein. These results suggest that certain SMN mutants can sequester the wild-type protein into inactive complexes. Molecular modeling of the SMN YG box dimer provides a structural basis for this dominant phenotype. These data demonstrate that important structural and functional features of the SMN YG box are conserved between vertebrates and invertebrates, emphasizing the importance of self-interaction to the proper functioning of SMN.

## Author Summary

Spinal Muscular Atrophy (SMA) is a prevalent childhood neuromuscular disease, which in its most common form causes death by the age of two. One in fifty Americans is a carrier for SMA, making this genetic disease a serious health concern. SMA is caused by loss of function mutations in the *survival motor neuron 1* (*SMN1*) gene. SMN is an essential protein and has a well-characterized function in the assembly of small nuclear ribonucleoproteins (snRNPs), which are core components of the spliceosome. To elucidate the phenotypic consequences of disrupting specific SMN protein interactions, we have generated a series of SMA-causing point mutations, modeled in *Drosophila melanogaster*. Using this system, we have shown that key aspects of SMN structure and function are conserved between humans and flies. Intragenic complementation analyses reveal the potential for dominant negative interactions between wild-type and mutant SMN subunits, highlighting the essential nature of the YG box in formation of higher-order SMN multimers. These results provide a basis for future studies investigating therapy targeted at restoration of functional SMN oligomers.

## Introduction

Proximal spinal muscular atrophy (SMA) is a common neuromuscular disorder, recognized as the most prevalent genetic cause of early childhood mortality [1]. SMA is characterized by degeneration of motor neurons in the anterior horn of the lower spinal cord, and progressive symmetrical paralysis. Coupled with this loss of motor function, SMA patients display severe atrophy of the proximal muscles. The onset of symptoms and their severity can vary, leading to an historical classification of SMA into three distinct subtypes [1]. More recently, clinicians have recognized that SMA is better characterized as a continuous spectrum disorder, ranging from severe (prenatal onset) to nearly asymptomatic [2].

Almost two decades ago, mutations in the *survival motor neuron 1* (*SMN1*) gene were shown to be causative for SMA [3]. The disease typically results from homozygous deletion of *SMN1*; however, a small fraction of SMA patients have lost one copy of *SMN1* and the remaining copy contains a point mutation [4]. The best characterized function for the ubiquitously expressed SMN protein is in the biogenesis of Sm-class small nuclear ribonucleoproteins (snRNPs), core factors of the spliceosome [5], [6]. In addition, SMN has been implicated in numerous other cellular activities, including axonal transport, neuronal pathfinding, formation and function of neuromuscular junctions, myoblast fusion and maintenance of muscle architecture [4], [7]–[11]. Despite this multitude of putative functions attributed to SMN, or perhaps because of it, the precise pathophysiological mechanisms that give rise to SMA are the subject of considerable debate. Seemingly straightforward questions of disease pathogenesis have yet to be definitively answered. For example, is SMA caused by a cell-autonomous reduction of SMN protein levels in motor neurons [12]–[16] or is it a more systemic defect involving other cell types [17]–[24]?

Irrespective of the question of *cellular* autonomy, the *molecular* etiology of SMA also remains unclear. Is the neuromuscular dysfunction seen in SMA patients caused by a loss of Sm-class spliceosomal snRNPs, ultimately leading to defects in pre-mRNA splicing? Or is it due to some non-canonical function of SMN and/or snRNPs? Experiments using animal models of severe SMA suggest that the pre-mRNA splicing defects observed in late-stage SMA animals are indeed tissue-specific [25], [26]. However, such deficits are only detectable later in the disease course, after the onset of neuromuscular dysfunction [27]–[29]. Complicating matters, SMN-deficient animals are developmentally delayed or arrested [22], [29]–[34], making the selection of properly staged control animals critical to the comparison of phenotypes. Moreover, alternative splicing of the SMN gene duplicate *(SMN2)* in humans or mouse models is another variable, creating a feedback loop [35], [36] that can negatively regulate SMN expression. In the fruitfly, the vast majority of *Smn* pre-mRNA transcripts are intronless (Flybase; [37]). Therefore, we set out to create an allelic series of *Drosophila* SMA models wherein we could specifically focus on SMN protein function, in the absence of other complicating factors.

## Results and Discussion

### Generation of *Drosophila* models of SMA patient mutations

**Figure 1:**
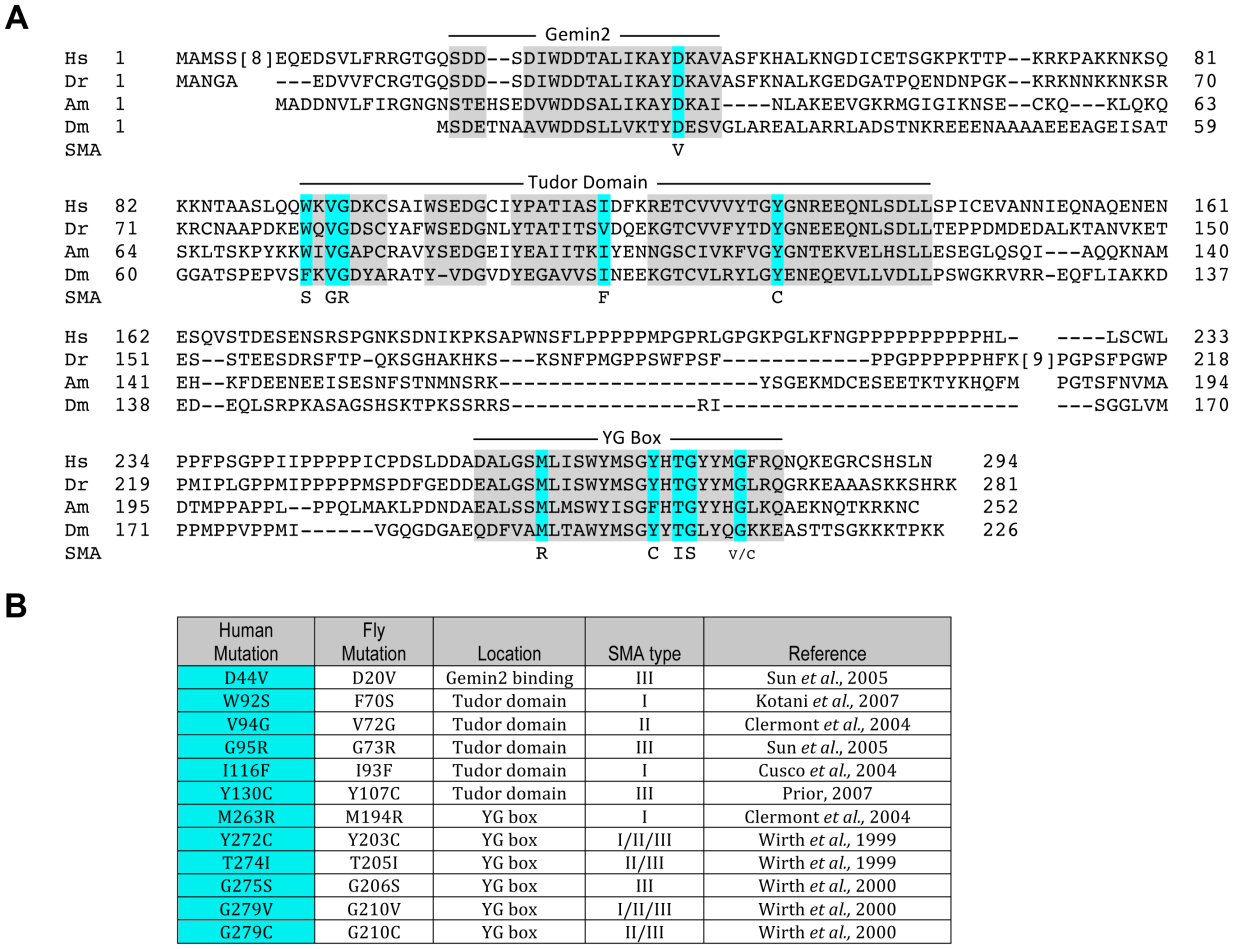
An allelic series of SMN missense mutations in *Drosophila*. (A) Alignment of SMN orthologues from *Homo sapiens* (Hs), *Danio Rerio* (Dr), *Apis Mellifera* (Am), and *Drosophila melanogaster* (Dm). Conserved regions, including the Gemin2 binding domain, the Tudor domain and the YG box of SMN, are shaded in gray. Residues known to be mutated in SMA patients are highlighted in aqua. SMA-causing missense residues (SMA) are listed below the highlighted residues. (B) Summary of the human SMA patient mutations that were modeled in *Drosophila*. These mutations affect all major domains of SMN, and encompass all three classes of SMA severity, with Type I being the most severe and Type III being the mildest form. When multiple SMA types are listed for a given mutation, this indicates that differing phenotypic severities were observed in patients bearing the same mutation.

To identify which functions of SMN are critical to the pathology of SMA, we aimed to create disease-relevant models that disrupt subsets of SMN interactions. Point mutations are useful in this context, as they can disrupt specific functions of multi-domain proteins, leaving other functions unaffected. To date, twenty-five different *SMN1* point mutations have been identified in SMA patients [4]. Many of these mutations are located at residues that are conserved between humans and insects (Fig. 1A). In this report, transgenic *Drosophila* bearing twelve of these SMA-causing point mutations were generated (Fig. 1B). The salient features of the *Smn* transgenic cassette have been previously described [28], including a 3X FLAG tag that was inserted immediately downstream of the ATG start codon. The transgenes were also expressed under control of the native *Smn* promoter and 3′ flanking sequences [28] and inserted at a landing site located within band 86Fb on chromosome 3R using the PhiC31 integrase system [38]. Note that all of the constructs (including an *Smn^WT^* control) were injected directly into embryos heterozygous for the *Smn^X7^* microdeletion, a null mutation [39] that was recombined with the appropriate PhiC31 landing site prior to injection (see Methods for details). Note that the *Smn^X7^* deletion removes the promoter region and the entire *Smn* open reading frame, leaving behind only 44 bp of the 3′ UTR [39]. Thus, this mutant produces no *Smn* mRNA.

As shown in Fig. 1, one of the mutations lies in the region responsible for Gemin2 binding, five are located in the Tudor domain, and six in the YG box oligomerization domain of SMN, thus mirroring the distribution of point mutations identified in SMA patients. For phenotypic analysis of the point mutants in our model, we crossed the transgenic flies (*Smn^X7^, Flag-Smn^Tg^*) with an *Smn^X7^* null allele to obtain flies that are homozygous null for endogenous *Smn* and hemizygous for the *Flag*-*Smn* transgene. We adopted this approach because human SMA patients that express *SMN1* missense mutations are also typically hemizygous and because we observed an improvement in the viability of the transgenic mutants when crossed to *Smn^X7^*, as compared to the self-cross. This latter finding indicates the presence of recessive alleles in the transgenic background that contribute to the phenotype of the homozygotes.

### SMA patient-derived mutations show a range of phenotypic severities in *Drosophila*

**Figure 2:**
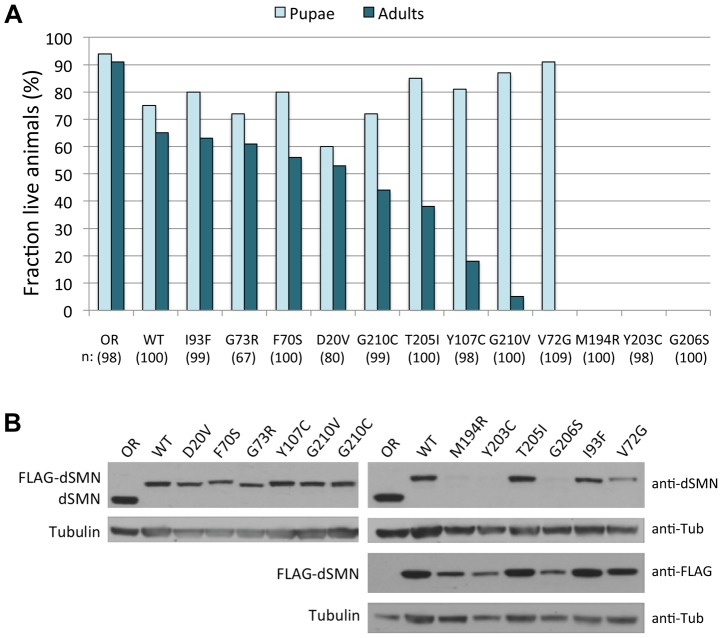
SMA patient-derived mutations show a range of life expectancies in *Drosophila*. (A) Viability analyses of SMA point mutations in *Drosophila*. *Smn^X7^/TM6.tb GFP* flies were crossed with each mutant line (*Smn^X7^,Smn^Tg^/TM6.tb GFP*) and hemizygous first instar mutant larvae (*Smn^X7/X7^, Smn^Tg/−^* where *Tg* stands for *transgene*) were collected and followed through development. Oregon-R (OR) animals served as controls. The data for each genotype are expressed as a fraction of pupae or adults over the total number of starting larvae (n), listed below each genotype. Expression of the WT transgene (*Smn^X7/X7^,Smn^WT/−^*) shows robust rescue of the null phenotype (∼67% adults). The mutants show a range in severity, from very severe (larval lethal), to intermediate (pupal lethal), to mild (adults). (B) Expression levels of *Smn* transgenes were examined by western blotting. Larval lysates from homozygous mutant lines (*Smn^X7/X7^,Smn^Tg/Tg^*), were probed with anti-dSMN or anti-FLAG antibodies, as indicated. The slower migrating bands represent the FLAG-tagged transgenic proteins and the faster migrating band corresponds to endogenous dSMN, present only in the OR controls. The mutant transgenes show similar levels of dSMN protein compared to *Smn^WT^* animals, with the exception of *Smn^M194R^, Smn^Y203C^* and *Smn^G206S^*, which show a significant reduction. Tubulin was used as a loading control.

When expressed in an *Smn^X7/X7^* null background, the hemizygous *Flag-Smn^WT^* construct showed robust rescue of adult viability (∼67%, see Fig. 2A), consistent with our previous findings using an *Smn^X7/D^* null background [28]. The degree of rescue achieved by expressing each of the different SMA point mutations varied (Fig. 2A). Three of the transgenes (*Smn^M194R^, Smn^Y203C^* and *Smn^G206S^*) failed to rescue the larval lethality of the null animals (Fig. 2A). In contrast, a second group of point mutants rescued adult viability to a degree roughly similar to that of the *Smn^WT^* transgene, including: *Smn^D20V^, Smn^F70S^* and *Smn^G73R^* and *Smn^I93F^*. A third group of mutants can be characterized as having intermediate phenotypes: *Smn^V72G^, Smn^Y107C^*, *Smn^T205I^*, *Smn^G210C^* and *Smn^G210V^*. This last category can be categorized as pupal-lethal, as the majority of these animals die prior to eclosion. The *Smn^G210C^* mutation produced a much milder phenotype (∼45% rescue to adulthood) compared to the *Smn^G210V^* mutation (∼5% rescue) despite the fact that they are located at the same residue. Interestingly, *Smn^V72G^* was the only mutation that rescued the larval lethality of the null animals but then also resulted in complete pupal lethality (Fig. 2A). Thus, when expressed in *Drosophila*, disease-causing *Smn* point mutations display a range in the age of symptomatic onset and in life-expectancy.

As shown previously [28], the amount of dSMN protein expressed from transgenes driven by the native *Smn* promoter and integrated at site 86Fb is several-fold lower than that of the endogenous gene (Fig. 2B). However, we observed robust rescue of the null phenotype in the presence of the *Smn^WT^* transgene, as these animals are both viable and fertile. A majority of the SMA alleles expressed equivalent levels of transgenic protein when compared to *Smn^WT^*. The exceptions were *Smn^M194R^*, *Smn^Y203C^* and *Smn^G206S^*, which showed a significant reduction in dSMN levels (Fig. 2B). Because the constructs were all inserted into the identical genomic location, and this same level of expression was observed in multiple independent transformants (Fig. S1), the reduction is most likely due to instability of the mutant proteins rather than differences in mRNA transcription. Furthermore, the very low levels of dSMN protein in the *Smn^M194R^*, *Smn^Y203C^* and *Smn^G206S^* animals are consistent with their severe, larval-lethal phenotypes.

### SMA-causing mutations are biochemically similar in human and *Drosophila*

**Figure 3:**
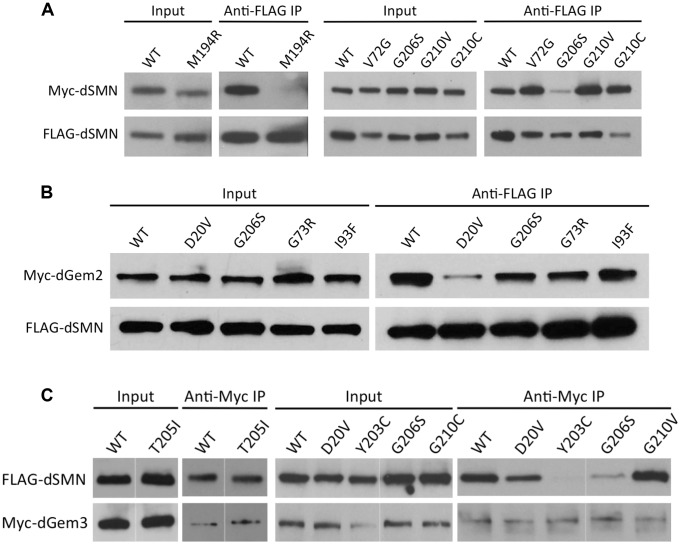
Interaction of SMN mutants with proteins involved in RNP assembly. (A) *Smn^M194R^* and *Smn^G206S^* are defective in a self-oligomerization assay. Note that oligomerization of *Smn^Y203C^* and *Smn^T205I^* was tested previously [28]. Lysates were prepared from cells co-expressing FLAG- and Myc-tagged versions of dSMN mutants or WT controls. Co-immunoprecipitation was performed with anti-FLAG antibody followed by western analysis with anti-Myc antibody to visualize the amount of Myc-tagged dSMN that co-precipitated with the FLAG-tagged protein. (B) dSMN(D20V) protein shows defective binding to dGem2. Immunoprecipitation was performed with anti-FLAG antibody followed by western analysis with anti-Myc antibody to visualize the amount of Myc-tagged dGem2. (C) dSMN(Y203C) and dSMN(G206S) are defective in their ability to bind dGem3. Immunoprecipitation of Myc-dGem3 was followed by probing with anti-FLAG antibody to visualize Flag-dSMN.

The ability of SMN to interact with itself [40], [41] is important for its function in RNP assembly [42], and mutations that disrupt SMN self-oligomerization are unstable *in vivo* [43]. We have shown previously that dSMN(Y203C) and dSMN(G206S) proteins are oligomerization defective [28]. We therefore tested dSMN(M194R) for its ability to self-interact. The mutant protein was N-terminally tagged with a 3X FLAG tag to distinguish it from the endogenous dSMN, and co-transfected into S2 cells (a *Drosophila* embryonic cell line) along with either a Myc-tagged dSMN(M194R) construct or a wild-type control. Immunoprecipitations were performed with anti-FLAG antibodies and the amount of co-precipitating protein was visualized by western blotting. As shown in Fig. 3A, dSMN(M194R) protein is also severely defective in its ability to self-oligomerize. Thus, the low levels of dSMN in *Smn^M194R^*, *Smn^Y203C^* and *Smn^G206S^* animals can be explained by the inability of these proteins to form higher-order SMN complexes. These observations are consistent with findings that the equivalent mutations in human SMN (*SMN^M263R^, SMN^Y272C^* and *SMN^G275S^*) are defective in self-oligomerization *in vitro* [40], [44], suggesting a high level of conservation between the structure-function relationships of *Drosophila* and human SMN.

Among the proteins that comprise the SMN complex in humans, collectively known as ‘Gemins,’ orthologues for Gemins2, 3, and 5 are the only genes that have been identified and validated in *Drosophila*. We and others [33], [45] have thus far failed to detect a biochemically stable interaction between dSMN and dGemin5 (dGem5), although dGem5 reportedly co-localizes with dSMN in various subcellular organelles [46]. To further establish whether dSMN can recapitulate the biochemical characteristics of SMA patient mutations, and to understand how these characteristics correlate with animal phenotypes, we analyzed the mutant dSMN proteins for their ability to bind dGem2 and dGem3 using a co-transfection assay.

The binding of dGem2 was reduced by a single *Smn* point mutation. As shown in Fig. 3B, dGem2 binding to dSMN(D20V) was reduced as compared to wild-type, but not eliminated. None of the other dSMN mutants showed any decrease in binding to dGem2 (Fig. 3B). This was not a surprising finding, as residue D20 lies within the known Gemin2 binding domain of SMN (Fig. 1A), and is consistent with experiments on its human counterpart, SMN(D44V) [47], [48]. Interestingly, *Smn^D20V^* flies display a mild phenotype, suggesting that the observed residual interaction with dGem2 is sufficient for its function *in vivo*. Indeed, SMA patients with the D44V mutation have been diagnosed with the mild form of SMA, type III [49].

We also analyzed the interaction of various Flag-tagged dSMN mutants with Myc-dGem3 by co-immunoprecipitation with anti-Myc antibodies. As shown in Fig. 3C, we found that binding of dGem3 was severely disrupted by two point mutations, *Smn^Y203C^* (*SMN^Y272C^* in humans) and *Smn^G206S^* (*SMN^G275S^*). Consistent with our findings, the human *SMN^Y272C^* mutation was previously shown to disrupt Gemin3 binding *in vitro* [40]. Notably, we found that the *Smn^T205I^* (*SMN^T274I^*) mutation, which lies between residues Y203 and G206, did not affect dSMN's ability to interact with dGem3 (Fig. 3C). This finding is consistent with previous observations that *SMN^T274I^* is active in snRNP assembly, an activity that also requires Gemin3 [42]. Strikingly, the phenotype of the *Smn^T205I^* animals was much less severe than that of either the *Smn^Y203C^* or *Smn^G206S^* mutants (Fig. 2A). Given that dSMN(T205I) and dSMN(G206S) proteins are both moderately defective in self-oligomerization ([28] and Fig. 3A), we attribute the more severe phenotype of the *Smn^G206S^* mutants to the inability of dSMN(G206S) to interact with dGem3. This interpretation is bolstered by the fact that null mutations in *Gemin3* were shown to destabilize dSMN *in vivo* [33]. In summary, these results further demonstrate that important biochemical properties of SMN are conserved between *Drosophila* and humans [24], [28], [45].

### *Smn* missense mutations display incomplete dominance

**Figure 4:**
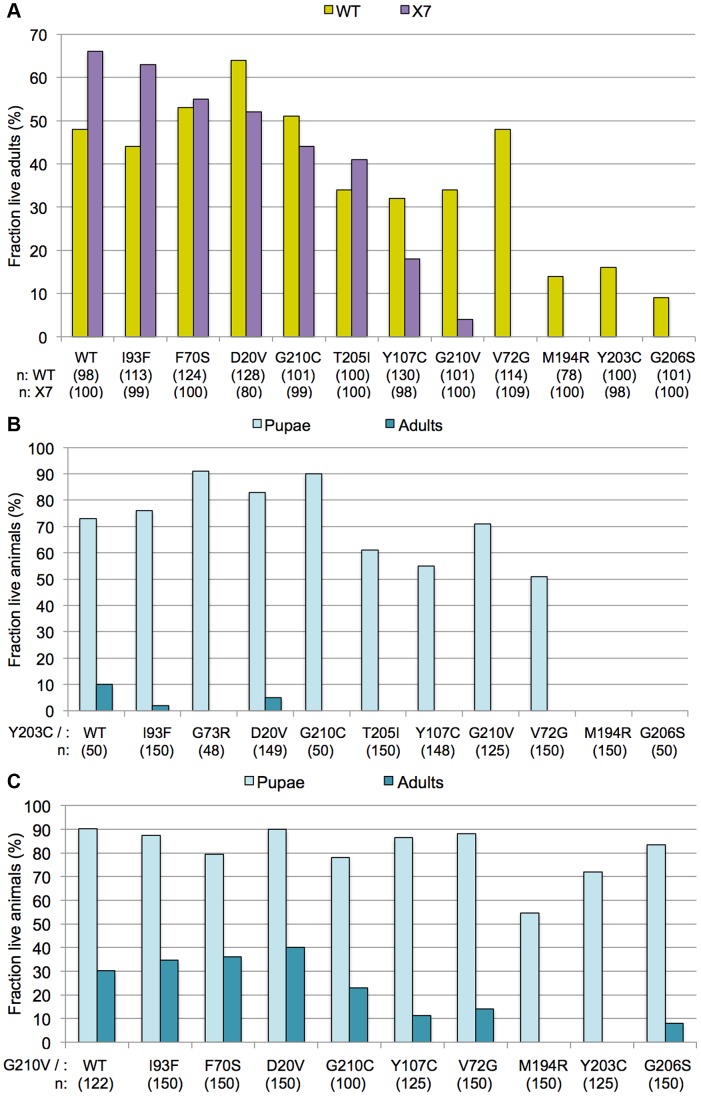
Incomplete dominance of *Smn* mutants. (A) Each of the *Smn* point mutant lines (*Smn^X7^,Smn^Tg^/TM6-tb.GFP*) was crossed to the wild-type (WT) rescue line (*Smn^WT^*,*Smn^WT^/TM6-tb.GFP*). For most mutations, this resulted in an intermediate level of rescue, between that observed when expressing the wild-type transgene alone versus that of the mutant transgenes alone. *Smn^X7/X7^*,*Smn^Tg/WT^* flies (WT) are shown in yellow and *Smn^X7/X7^*,*Smn^Tg/−^* flies (X7) are shown in purple. Genotypes of the various transgenes are listed along the X-axis. Larval progeny were followed through development and the number of eclosing adult flies was measured and expressed as a fraction of the total number of starting animals (n) for each genotype. (B) The *Smn^X7^,Smn^Y203C^* mutants were crossed with each of the other transgenic lines, both mutant (*Smn^X7^,Smn^Tg^*) and wild-type (*Smn^X7^,Smn^WT^*). Larval progeny were followed through development and the number of pupae and adults was measured and expressed as a fraction of the total number of starting animals (n) for each genotype. (C) The *Smn^X7^,Smn^G210V^* mutants were crossed to the other alleles, as described in panel B.

To further characterize the *Smn* point mutants, we crossed each of them to the wild-type (WT) rescue line, *Smn^WT^*. Since SMA patients display a recessive mode of inheritance, we expected that the *Smn^WT^* transgene would rescue organismal viability in the missense mutant backgrounds (genotype: *Smn^X7/X7^,Flag-Smn^WT/Mut^*) to roughly the same extent as it did in the hemizygous rescue line (*Smn^X7/X7^,Flag-Smn^WT/−^*). However, for many mutations we observed an intermediate level of rescue, between that observed when expressing the wild-type transgene alone versus that of the mutant transgenes alone (Fig. 4A). This dominant phenotype was most evident when the wild-type transgene was expressed in combination with the three most severe YG box alleles (*M194R, Y203C* and *G206S*). For example *Smn^X7/X7^,Flag-Smn^WT/Y203C^* animals displayed a much lower eclosion frequency (Fig. 4A) than did either of the two controls, *Smn^X7/X7^,Flag-Smn^WT/−^* (hemizygotes) or *Smn^X7/X7^,Flag-Smn^WT/WT^* (homozygotes). Importantly, expression of the wild-type *Smn* transgene in combination with the *M194R, Y203C* or *G206S* point mutants rescued the larval lethality associated with expression these alleles on their own (Fig. 4A). Thus, these dominant phenotypes are intermediate in their severity.

We note that *Smn^WT/WT^* homozygotes (obtained by an intercross of two different founder lines) showed a somewhat lower eclosion frequency (∼50%) than did the hemizygotes (∼70%). These findings suggest that second-site recessive alleles may contribute to the decreased fitness of the homozygotes and that outcrossing to a ‘clean’ *Smn^X7^* chromosome ameliorates these effects. Moreover, the presence of second site recessives may also affect the results of the intragenic complementation analyses (Fig. 4A), as the transgenes were all inserted into the same genetic background. However, when co-expressed with *Smn^WT^*, the phenotypic severities observed for the point mutant lines correlated with the degree of rescue when expressed alone. For example, *Smn^G210V^* had a less severe phenotype (pupal lethal) than *Smn^G206S^* (larval lethal), when expressed alone. When co-expressed with the wild-type transgene, *Smn^G210V/WT^* flies also showed a milder phenotype than did *Smn^G206S/WT^* (Fig. 4A). There was one exception to this rule; *Smn^V72G^* displayed an early pupal-lethal phenotype when expressed alone, but had an eclosion frequency comparable to that of the mild mutations when co-expressed with *Smn^WT^*. In summary, complementation crosses with the mutant and wild-type *Smn* transgene display intermediate phenotypes.

Using *Smn^Y203C^* and *Smn^G210V^* as test cases, we carried out additional complementation crosses to each of the other point mutant lines. As shown in Fig. 4B, co-expression of *Smn^Y203C^* with the other transgenes had a negative effect on adult viability. When crossed to *Y203C*, alleles that showed strong rescue of the null phenotype when expressed alone (e.g. *WT, D20V, G73R*, or *I93F*) either completely failed to eclose as adults or did so at very low frequencies. However, this dominant effect was not fully penetrant, as a majority of the trans-heterozygous animals developed beyond larval stages and died as pupae (Fig. 4B). The only exceptions were crosses between *Y203C* and the other two severe mutations (*M194R* and *G206S*), which continued to die as larvae. *Smn^G210V^* co-expression with the other transgenes had a positive effect on pupation of flies expressing the severe mutations (Fig. 4C). Overall, when crossed to *G210V*, adult viability decreased. Crosses between *G210V* and either *V72G* or *G206S* were the only exceptions, with a small fraction of flies eclosing. This is in contrast to the pupal lethality observed when *V72G* was expressed alone and the larval lethality of *G206S*. Thus the intermediate phenotypes observed in these complementation crosses provide a clear example of the genetic principle of incomplete dominance.

### Severe YG box mutants retain the ability to interact with wild-type SMN and display a more pronounced phenotype than *Smn* null animals

**Figure 5:**
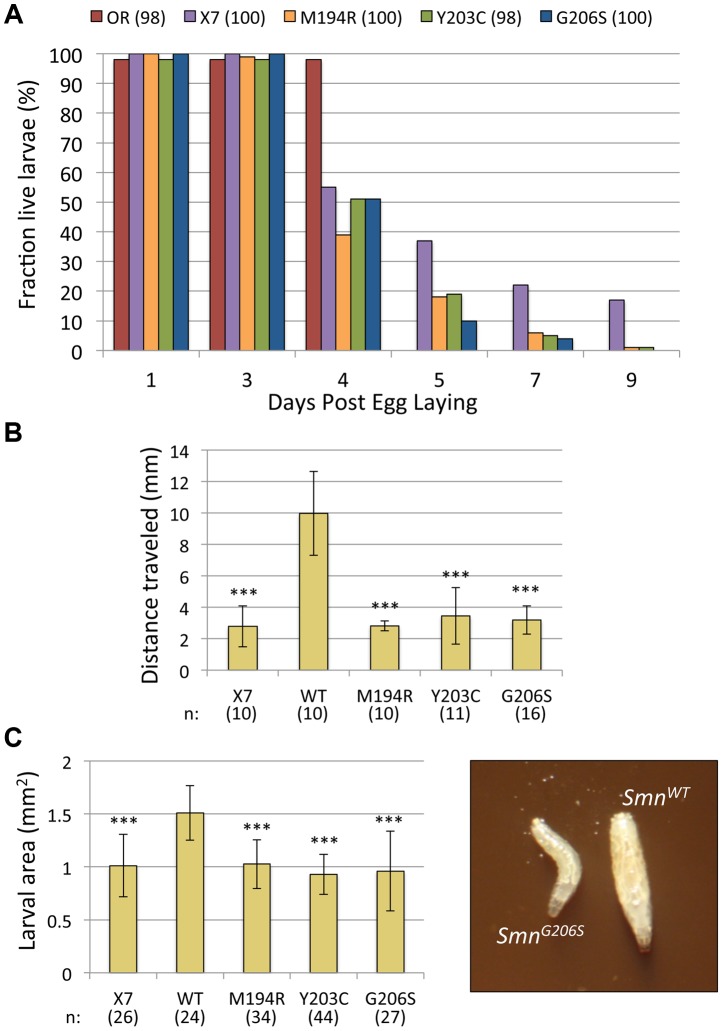
Phenotypic characterization of three severe YG box mutants. (A) Viability of larvae hemizygous for the *Smn^M194R^, Smn^G206S^ and Smn^Y203C^* mutations was assayed. The graph tracks survival of approximately100 larvae (n) for each genotype (*Smn^X7/X7^,Smn^Tg/−^*), along with homozygous null mutant (*Smn^X7^/Smn^X7^*) and Oregon-R (OR) controls, over time. All three missense mutants die earlier than the null animals, suggesting a dominant negative effect. Note that by day 5 all of the wild-type control larvae have pupated. (B) Graph of average distance traveled over 20 s after stimulation with a needle (n>10 larvae were scored for each genotype). Larval movement was impaired for *M194R, Y203C and G206S* larvae, relative to *WT* transgene controls (P<1.3×10^−5^). (C) Graph of overall larval size, as measured by area (in mm^2^). For each genotype, n>24 larvae were scored (P<1.8×10^−7^). For illustration, an image of two representative larvae (*Smn^WT^* and *Smn^G206S^*) is shown.

The most severe *Smn* point mutations in our collection (*M194R*, *Y203C* and *G206S*) all map to the YG box self-oligomerization domain (Fig. 1A), which is the most well-conserved region of SMN. Consistent with their YG box location, these three alleles are defective in self-interaction assays (Fig. 3A) and do not form stable SMN complexes *in vivo* (Fig. 2B). By such criteria, these three alleles would normally be considered to be protein nulls, yet their phenotype is actually more severe than that of the null allele, *Smn^X7/X7^*. Indeed, these larvae all died by ∼8–9 days after hatching, whereas ∼20% of the null mutants were still alive at this time point (Fig. 5A). The *M194R*, *Y203C* and *G206S* larvae are similar in size to *Smn* null mutants and are noticeably smaller and less active their *Smn^WT^* counterparts (Figs. 5B and 5C). These latter observations are consistent with previous analyses of *Smn* null mutants [19], [31], [33]. The long-lived larval phenotype displayed by the *Smn* null animals [28], [33], but not by the severe point mutants (Fig. 5A), suggests that zygotic expression of the oligomerization-defective point mutations inhibits the function of the endogenous Gemins and/or the maternally contributed (wild-type) dSMN.

**Figure 6:**
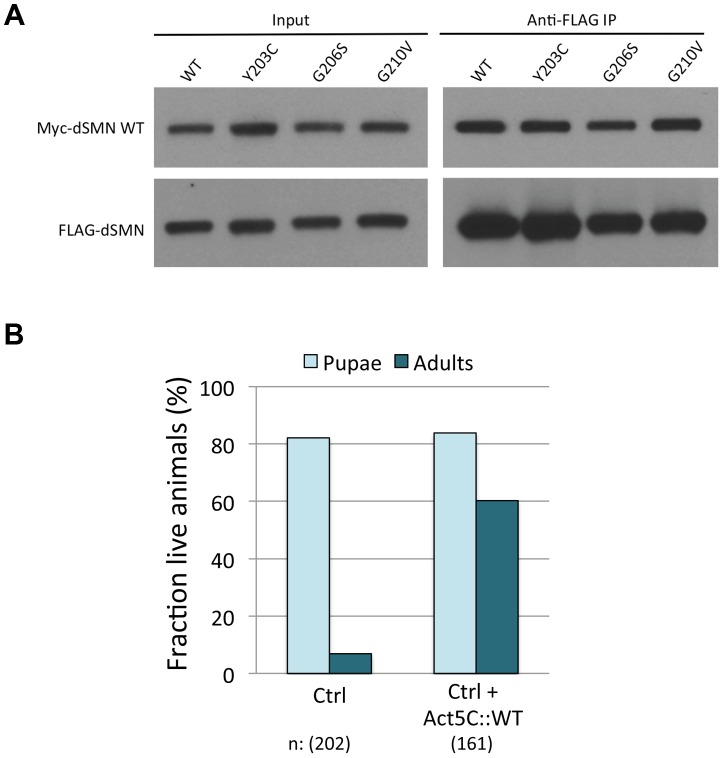
Severe YG box mutants retain the ability to interact with wild-type SMN and overexpression of WT Flag-dSMN rescues their dominant negative effect. (A) Self-oligomerization defective YG box mutants are still able to interact with WT dSMN. Lysate from cells co-transfected with Myc tagged WT *Smn* and FLAG tagged mutant protein were immunoprecipitated with anti-FLAG antibody. The amount of co-precipitating Myc-WT dSMN was visualized by western blotting. A variety of FLAG-tagged dSMN mutants including *Y203C, G206S* and *G210V* were able to pull down Myc-WT dSMN. (B) The wild-type dSMN protein was overexpressed in an *Smn^Y203C/WT^* background using a *UAS::Flag-Smn^WT^* transgene driven by *Actin-5C::GAL4*. Control larvae (*UAS::Smn^WT^/CyoGFP; Smn^X7^*,*Smn^WT^*/*Smn^X7^,Smn^Y203C^*, labeled Ctrl) and overexpression larvae (*UAS::Smn^WT^/Actin5C::Gal4; Smn^X7^*,*Smn^WT^*/*Smn^X7^,Smn^Y203C^*, labeled Ctrl+Act5C::WT) were followed over development and the numbers of pupae and adults were measured. Overexpression of dSMN ameliorates the dominant negative effect of the *Smn^Y203C^* transgene by increasing the number of eclosing adults from ∼6% to ∼60%.

Despite the fact that the severe YG box mutants are unstable and fail to self-interact, we reasoned that the dominant negative phenotype seen upon co-expression of wild-type and mutant dSMN suggests that these two proteins might form heterodimers. We tested this idea by co-transfecting differentially tagged wild-type and mutant dSMN constructs and measuring the amount of co-precipitated protein. We assayed *Y203C, G206S*, *G210V* (Fig. 6A) along with a number of other mutant Flag-dSMN constructs, and found that they were able to pull down wild-type Myc-dSMN. Consistent with these findings, Martin et al. [44] recently showed that human SMN YG box mutants *M263R, Y272C* and *G275S* (corresponding to *M194R*, *Y203C* and *G206S* in flies) were completely unable to form dimers or higher-order multimers *in vitro*, as measured by multi-angle light scattering (SEC-MALS). In contrast, a relatively mild YG box mutation, *T274I* (*T205I* in flies), was only partially defective in self-interaction [28], [40], [44], [50]. We conclude that the dominant negative interaction of *Smn* alleles is not limited to zygotically expressed protein, but extends to the maternal contribution as well. Moreover, these findings show that important structural and functional features of the SMN YG box are conserved between vertebrates and invertebrates.

### Overexpression of *Flag-Smn^WT^* suppresses SMN dominant negative interactions

It is important to note that the dominant effect of expressing the *M194R*, *Y203C* and *G206S* transgenes was not observed when these alleles were carried over a balancer or a wild-type third chromosome. *Smn* transgenes integrated at the 86Fb landing site express relatively low levels of Flag-dSMN, compared to the endogenous gene located at 73A9 (Fig. 2B). One interpretation of these results is that high levels of wild-type dSMN expression from the endogenous gene can squelch the activity of the mutant transgenic protein. Alternatively, the observed decrease in viability of the heteroallelic *Smn* combinations could be due to second site recessive mutations, as the transgenes were all inserted into the same genetic background. To explicitly address this question, we over-expressed wild-type Flag-dSMN from a transgene located on the second chromosome, using the UAS-Gal4 system [51]. Next, we measured eclosion frequencies of *UAS-Flag-Smn,act5C-Gal4; Smn^X7/X7^,Flag-Smn^WT/Y203C^* transgenic rescue animals versus those that lack the *act5C-Gal4* driver. As shown in Fig. 6B, overexpression of WT Flag-dSMN increased the eclosion frequency from ∼6% to ∼60%. Equivalent results were obtained with a *tubulin-Gal4* driver. Thus, we can fully rescue the dominant negative effect of the heteroallelic *Smn^Y203C/WT^* transgenes by overexpressing WT dSMN, demonstrating that the decrease in viability of these animals is not due to second site recessives. We therefore conclude that severe *Smn* YG box mutations can act in a dominant fashion, given that our transgenic alleles are expressed at relatively low (but equivalent) levels, the observed dominant behavior of the severe YG box mutants could also be viewed as creating a haploinsufficient condition, titrating away wildtype SMN monomers and/or Gemin components. In either event, the ‘squelching’ experiment (Fig. 6B) is important because it suggests that overexpression of full-length SMN protein (e.g. from *SMN2*) may be an effective therapy for SMA patients bearing severe *SMN1* point mutations.

### Structural basis for the YG box dominant negative phenotype

**Figure 7:**
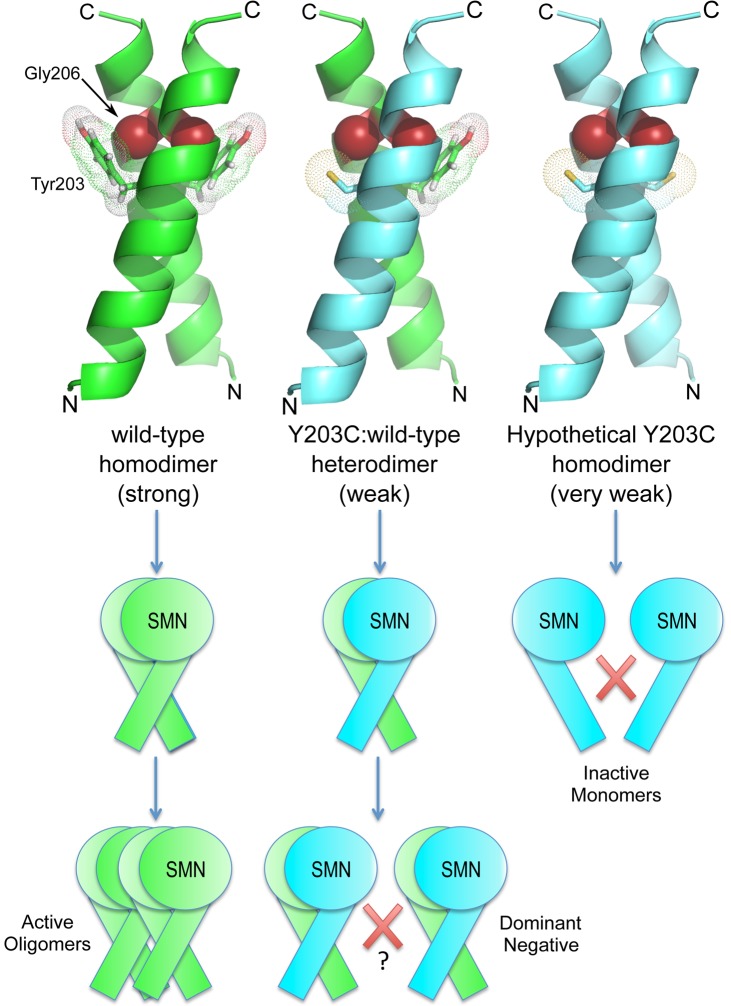
*SMN^Y203C^* is predicted to disrupt an intermolecular Y-G contact, weakening or preventing oligomerization. A combination of experimental analysis and structural modeling of *Drosophila* SMN dimers reveals that wild-type homodimers (left column) can make two Y-G contacts and are able to form higher-order structures (active SMN oligomers). In contrast, Y203C homodimers (right) are unable to make these contacts and do not dimerize (inactive SMN monomers). Interestingly, Y203C:WT heterodimers (middle) make only one Y-G contact but the loss of one interaction does not prevent dimerization. These findings suggest that the dominant negative activity of Y203C is due to an inability of the heterodimers to form active, higher-order oligomers.

Within the SMN YG box dimer [44], conserved tyrosine and glycine residues are thought to form a network of intersubunit interactions, wherein each tyrosine side chain packs against the main chain atoms of the of the i+3 glycine on the opposing helix. As illustrated in Fig. 7, molecular modeling of *Drosophila* SMN dimers reveals that the Y203C mutation is predicted to disrupt this intermolecular Y-G contact. Wild-type homodimers can form two such interactions, whereas Y203C:WT heterodimers can form only one of them; Y203C homodimers are incapable of making these contacts (Fig. 7). Similarly, mutation of G206 to a bulkier serine is predicted to have a disruptive effect. Most important, the Y203-G206 contact occurs precisely at the point at which the two helices cross, so this interaction is expected to be most critical for stability of the SMN dimer.

Interestingly, the highly conserved threonine residue present within the _203_YxTG_206_ motif does not participate directly in the dimer interface [44]. Consistent with this observation, we found that the *T205I* mutant interacts with dGem3, whereas the *Y203C* and *G206S* constructs do not. These results suggest that dGem3 interacts with a multimeric form of SMN.

Because the human SMN crystal structure [44] does not contain any information regarding residues that are located upstream of M263 (M194 in the fruitfly), we are currently unable to create an accurate molecular model of the M194R mutation. However, it is interesting to note that constructs containing the proximal N-terminal region of human SMN (i.e. residues 252–294 instead of 263–294) have a strong tendency to form higher-order structures, primarily tetramers and octamers [44]. Taken together with the relative instability of dSMN(M194R) protein (Fig. 2B), its inability to self-interact in cultured cells (Fig. 3A), and the severe larval-lethal phenotype of this mutation (Fig. 4), these results support the view that formation of higher-order multimers of SMN is important for its function *in vivo* (Fig. 7).

### Using the *Drosophila* system to assay SMN protein function

Gaining crucial insight from simpler model organisms is a proven strategy for unraveling complicated biological questions. Using a single point mutation (*Smn^T205I^*), we recently showed that the larval locomotion and adult viability defects associated with loss of SMN can be uncoupled from the snRNP assembly function of SMN [28]. More specifically, we found that complete loss of SMN did not result in appreciable changes in the splicing of minor intron genes [28], [29], thus arguing for a non-splicing mechanism for SMA etiology. In this report, we have conducted genetic and biochemical characterization of eleven additional *Smn* mutants. We show that this allelic series captures a range of phenotypic severities, further suggesting a high level of conservation between the human and fruitfly SMN proteins and related pathways.

The complex organization and polymorphic nature of the two human *SMN* genes complicates the analysis of SMA patient phenotypes. Indeed, *SMN2* copy number variation is the best known modifier of SMA, potentially masking the phenotype of *SMN1* point mutants [4]. However, SMN2 copy number is not always predictive of SMA disease severity [52]. Thus, developing an animal model that is an accurate predictor of SMN protein function is an important goal.

We find good correlation between the biochemical properties of the YG box mutants and their phenotypic severities. Patients bearing point mutations in *SMN1* are relatively rare, and genotypic information (*SMN2* copy number) is often not available. Such is the case for the only patient reported to bear a *G275S* mutation (*G206S* in flies), who presented with a mild form of SMA but *SMN2* copy number was not determined [53]. Biochemically speaking, *G275S* should be a severe mutation, as this mutant is unable to form oligomers [44]. Consistent with these findings, *G206S* is a severe mutation in the fly and fails to bind Gemin3 (this work). Similarly, four human patients bearing the *T274I* mutation presented with intermediate forms of SMA, yet they had only a single copy of *SMN2* [53], [54]. Given that the *T274I* mutant was shown to be active in Sm core assembly [42], the milder human phenotype was perhaps to be expected. Concordantly, the corresponding fly mutant, *T205I*, also displays an intermediate phenotype ([28]; this work). The same is true for *D44V* (*D20V*), which is located in the Gemin2 binding domain, and presents with a relatively mild phenotype in both humans and flies.

In contrast, the phenotypes of the Tudor domain mutants were harder to compare. Of the five mutations tested, two of them (*F70S* and *I93F*) were not as severe as one would have predicted from the human data (*W92S*, [55]; *I116F*, [56]). Using an S2 cell co-transfection assay similar to that in Fig. 3, we screened the entire panel of *Smn* mutants for their ability to bind to SmD1 and found no appreciable differences. Previous results suggest that *Drosophila* cells are less sensitive to the methylation status of Sm proteins than are human cells [57]–[59]. Given that the Tudor domain is thought to be a methyl-binding module, perhaps *Drosophila* are more tolerant to mutations in this region of SMN. However, we previously identified a synthetic lethal genetic interaction between *Smn* and *Dart5* (the fruitfly orthologue of the arginine methyltransferase, *PRMT5*; see [59]), so methylation of Sm proteins (or other SMN interactors) may not be completely dispensable in flies. In the absence of more quantitative biochemical assays of SMN function (particularly for the Tudor domain mutants), we are currently unable to make good correlations between genotype and phenotype. Thus, additional efforts in this area will be needed.

Additional mutations, those that are patient-derived as well as those that are predicted by ultrastructural studies, should greatly aide future investigations of the SMN YG box oligomerization motif by providing an all-important organismal readout. Moreover, additional phenotypic analyses (longevity, locomotion, flight, neuromuscular development, etc.) particularly of adult animals, should provide quantitative measures of differences between the weaker *Smn* alleles. Correlating this information along with proteomic and RNomic analyses of these alleles will provide important data on SMN function and SMA etiology.

## Materials and Methods

### Fly stocks

All stocks were cultured on molasses and agar at room temperature (24±1°C) in half-pint bottles. Oregon-R was used as the wild-type allele. The *Smn^X7^* microdeletion allele was a gift from S. Artavanis-Tsakonis (Harvard University, Cambridge, USA). This deficiency removes the promoter and the entire SMN coding region, leaving only the final 44 bp of the 3′ UTR [39].

### Larval size and locomotion

Control and mutant larvae (73–77 hrs post egg-laying) were imaged at 10× magnification with a stereo dissection microscope (Leica) at 10× magnification. Images were captured with a digital camera and larval outlines were traced. Total pixel area was calculated using ImageJ software and measurements converted to square millimeters. For larval locomotion, *Smn* control and mutant larvae (73–77 hours post egg-laying) were placed on 1.5% agarose molasses plates and prodded with a needle to stimulate movement. After 20 s an image was recorded (7× magnification) and the tracks were traced and total distance traveled was measured using ImageJ software. P-values were generated using a 2-tailed student's t-test, assuming unequal variance.

### Rescue constructs

As described in Praveen et al. [28], a ∼3 kb fragment containing the entire *Smn* coding region was inserted into the pAttB vector [38]. A 3× FLAG tag was inserted immediately downstream of the dSMN start codon. Point mutations were introduced into this construct using Quickchange (Invitrogen) site-directed mutagenesis according to manufacturer's instructions. The transgenes were injected directly into embryos heterozygous for the *Smn^X7^* microdeletion [39] that was recombined prior to injection with the 86Fb PhiC31 landing site (Bloomington Stock Center, IN, USA). The injections were performed by BestGene Inc. (Chino Hills, CA).

### Tissue culture and transfections

S2 cell lines were obtained from the *Drosophila* Genome Resource Center (Bloomington, IL). S2 cells were maintained in Express Five SFM (Gibco) supplemented with 1% penicillin/streptomycin and 9% L-glutamine. Cells were removed from the flask using a cell scraper and passaged to maintain a density of approximately 10^6^–10^7^ cells/mL. S2 cells were transferred to SF900 SFM (Gibco) prior to transfection with Cellfectin II (Invitrogen). Transfections were performed according to Cellfectin II protocol in a final volume of 3 mL in a T-25 flask containing 5×10^6^ cells that were plated one hour before transfection. The total amount of DNA used in transfections was 2.5 ug.

### Immunoprecipitation and western blotting

Larval lysates were prepared by crushing animals in lysis buffer (50 mM Tris-HCl [pH 7.5], 150 mM NaCl, 1 mM EDTA, 1% NP-40) with 1× protease inhibitor cocktail (Invitrogen) and clearing the lysate by centrifugation at 13,000 RPM for 10 min at 4°C. S2 cell lysates were prepared by suspending cells in lysis buffer (50 mM Tris-HCl [pH 7.5], 150 mM NaCl, 1 mM EDTA, 1% NP-40) with 10% glycerol and 1× protease inhibitor cocktail (Invitrogen) and disrupting cell membranes by pulling the suspension through a 25 gauge needle (Becton Dickinson). The lysate was then cleared by centrifugation at 13,000 RPM for 5 min at 4°C. Western blotting on lysates was performed using standard protocols. Rabbit anti-dSMN serum [28] was affinity purified. For Western blotting, dilutions of 1 in 2,500 for the affinity purified anti-dSMN, 1 in 10,000 for anti-α tubulin (Sigma), 1 in 10,000 for monoclonal anti-FLAG (Sigma), 1 in 10,000 for polyclonal anti-Myc and 1 in 5000 for monoclonal anti-Myc (Santa Cruz) were used. Anti-FLAG antibody crosslinked to agarose beads (EZview Red Anti-FLAG M2 affinity gel, Sigma) or anti-Myc antibody crosslinked to agarose beads (Sigma) were used to immunoprecipitate FLAG and Myc tagged proteins from cells.

### Structural modeling

A structural model of a *Drosophila* SMN YG-box dimer was generated by replacement of side chains in the Martin et al. [44] structure (pdb code 4GLI) with those that differ in the *Drosophila* SMN sequence. For each substitution, the new side chain rotamer was adjusted to be identical to that found in the human structure. No changes to the main chain conformation were made and no steric clashes were observed in the final model.

## Supporting Information

Figure S1 Flag-dSMN is expressed at similar levels in multiple independent transformants when inserted at the same genomic location. (A) Similar levels of Flag-dSMN expression are obtained in multiple independent transformants of the same SMN missense mutation inserted at the same genomic location (86Fb). Similar levels of expression between independent transformants are observed with all mutations, regardless of the severity of the phenotype in which they result. Lysates were prepared from flies expressing Flag-tagged versions of dSMN mutants. Anti-FLAG antibody was used to visualize the amount of Flag-dSMN. Tubulin was used as a loading control. (B) Levels of dSMN from an *Smn^WT^* transgene inserted at different genomic locations (86Fb, 65B2, and 68A4) vary depending on the insertion site. Independent transformants of 65B2 and 68A4 insertions are shown. Higher levels of dSMN expression are achieved using the 86Fb insertion site.
(PDF)
