# Supplementary figures and images for: SMA-Causing Missense Mutations in Survival motor neuron (Smn) Display a Wide Range of Phenotypes When Modeled in Drosophila
Source: PLoS Genet. 2014 Aug 21;10(8):e1004489. doi: 10.1371/journal.pgen.1004489 (PMC4140637; doi:10.1371/journal.pgen.1004489)

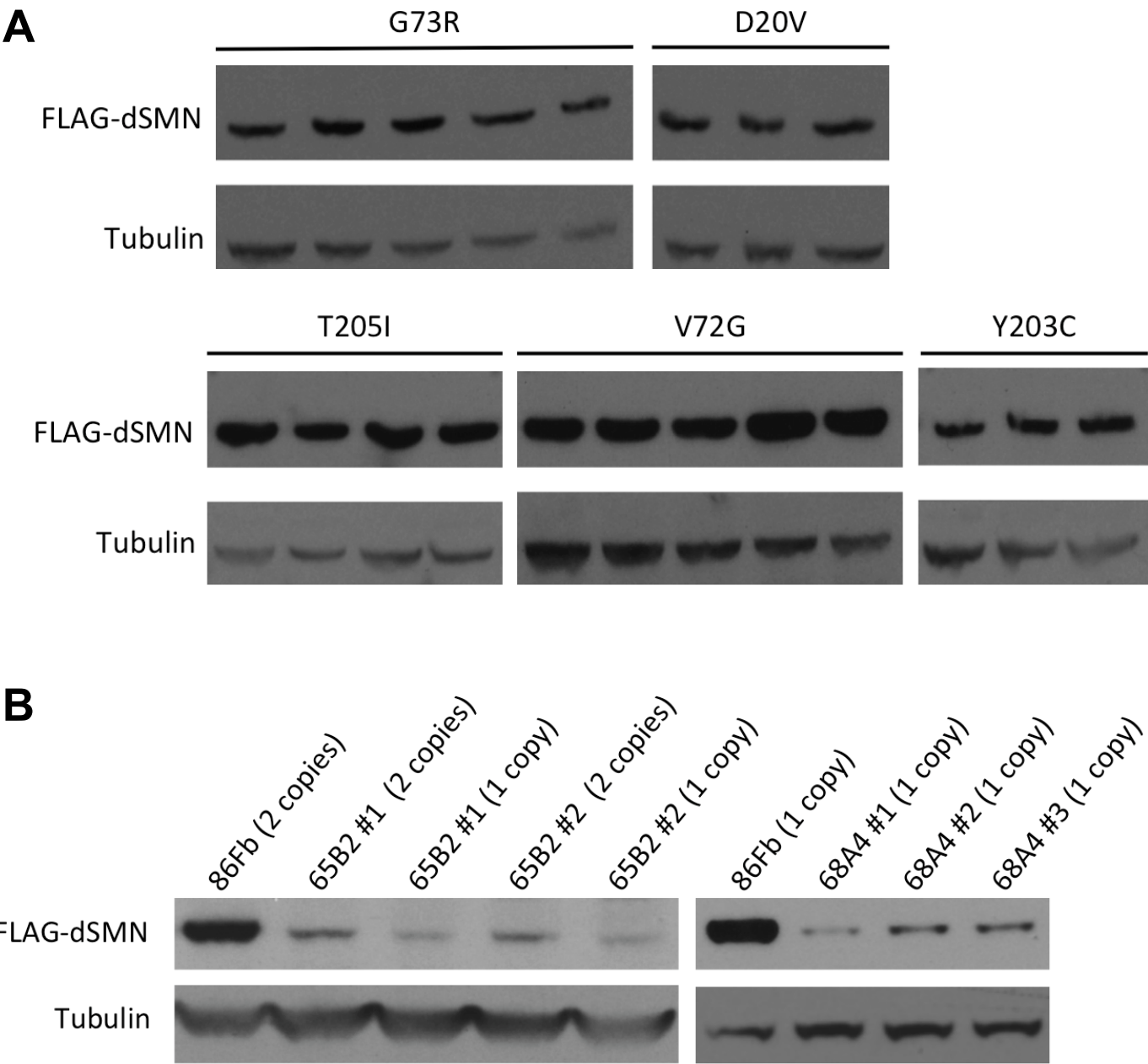

Supplement: Figure S1 — Flag-dSMN is expressed at similar levels in multiple independent transformants when inserted at the same genomic location. (A) Similar levels of Flag-dSMN expression are obtained in multiple independent transformants of the same SMN missense mutation inserted at the same genomic location (86Fb). Similar levels of expression between independent transformants are observed with all mutations, regardless of the severity of the phenotype in which they result. Lysates were prepared from flies expressing Flag-tagged versions of dSMN mutants. Anti-FLAG antibody was used to visualize the amount of Flag-dSMN. Tubulin was used as a loading control. (B) Levels of dSMN from an SmnWT transgene inserted at different genomic locations (86Fb, 65B2, and 68A4) vary depending on the insertion site. Independent transformants of 65B2 and 68A4 insertions are shown. Higher levels of dSMN expression are achieved using the 86Fb insertion site. (PDF) [file pgen.1004489.s001.pdf]
